# Alas! Poor Yorick

**Published:** 1886-10

**Authors:** 


### ALAS ! POOR YORICK !

DOUBTLESS, ere this, Dr. Billings—the brilliant "exception to the rule"—is timid about opening the medical journals. His absurd statements made before the British Medical Association about malaria and science being antagonistic have brought down upon him a shower of invective and ridicule. It seems that his aim was to create the impression that the medical profession of the South and West are ignorant, half educated, not "scientific," as a kind of a half-way justification of his course in the original organization of the International Medical Congress as proposed by him—the omission of members from those sections ;—and he went at it in a roundabout way. "Malaria" was dragged in as a kind of pretext for saying that there is no "science" in the great Mississippi valley.

But his plan was an unfortunate one, and has laid him open to severe ridicule. He asserts that "malaria and science are antagonistic;" the inference being that science exists only where there is no malaria ; and as he evidently intended to create the belief that all the science is up North, or " down East," he overlooked the fact that his map showed an abundance of absence of malaria—ergo—presence of science away up in the great Northwest, the Territories and Utah ; and also, where, according to Bartholow, there is dense ignorance—in southern Illinois. He and Dr. Bartholow should have compared notes better, for, as it stands, Billings insists, according to his map, that lower Illinois in immensely scientific.

Dr. Davis, in the *Journal of the American Medical Association* (Sept. 25), treats Billings and his "notable address" in a serio-comic vein that is exquisite—irresistable. His commentary on the map and the address is worthy of a Juvenal or a Dickens. After pointing out the existence of the greater part of the science of the medical profession of

America, away up in the great Northwest—up amongst the Rocky Mountains, and down in Lower California, and in Utah, according to this "map"—he says:

"Reasoning on these lines, and looking at the Utah part of the malarial map, one might be inclined to formulate some such law as 'Malaria and Polygamy are Antagonistic;' for there is a great deal of polygamy in Utah, but very little malaria; and while there is much malaria in the Mississippi valley, there is no polygamy; and this is further borne out by the fact that while the Sultan of Turkey has many wives, he is not subject to malaria. Now, then, if malaria and science are antagonistic, malaria and polygamy antagonistic, malaria and cancer antagonistic, and cancer and science synergistic, does it not follow that polygamy and science are synergistic? Have we not here a key to the great problem of what to do with the Mormons? Let the Government send half a dozen bactero-therapists to Utah, each armed with cultivations of the bacillus malariæ. * * * * Yet, if malaria and science *are* antagonistsc, there is still a gleam of hope—one way out of the difficulty; and but one. We regard it as fortunate in medicine, when we find something antagonistic to something else, which is dangerous; and if malaria be antagonistic to cancer, polygamy and science, the converse must be true. Here, then, we have a prophylactic indication; instead of planting eucalyptus trees, which must take some years to grow; instead of draining swampy lands, which requires a vast amount of money, let us transplant full grown cancer patients, polygamists and scientists, to our malarial districts, in such numbers that the malarial microbes must inevitably be overcome and driven from the country. Let a great cry go up from the Mississippi valley and other malarial districts for an influx of *cancer patients from the North and Northeast*, and *scientists from Idaho, Utah and Nevada.*" [Italics ours.]

The irony in that last sentence is exquisite. Dr. Davis insists that Billings meant *cancer*, and not science; that *cancer* is the only thing enjoyed by the East, not possessed by the South; as he has shown *by the "map"* that the "science" predominates in the Rocky Mountain region. (In this, Billing's reminds us of the little boy who boasted that "they" had something at "their" house, which none of the others had, and when asked what it was, triumphantly answered "a dead grand-ma.")

Dr. Davis concludes his masterly article in these telling words. Those who have been favored with seven immense volumes of "The Index Catalogue of the Surgeon General's Office" will not fail to see the point:

"And if the remedies prove potent against the evil, then we will see that science and malaria are really antogonistic; we will see how great results may spring from incidental suggestions made in a foreign country; and we will also see

'How *Index*-learning turns no student pale,
Yet holds the eel of science by the tail.'"

Poor Billings! With the whole of the Southern and Western Journals turned against him—and the *Journal of the American Medical Association* making fun of him, he must feel that he has made an unenviable notoriety; and, doubtless, he wishes that he had not been "the exception to the rule," but had followed the "old Scotch maxim," which he quotes: "That which you don't know, tell that not to any one."

It only remains now for some medical Thomas Nast to carricature him in the act of holding an eel labled "science" firmly by the tail, while a large number of eels, having eluded the grasp of the people in the Mississippi valley, are making tracks for the mighty East. (With a pile of *Index* catalogues in the foreground.)

